# Adaptation to a novel family environment involves both apparent and cryptic phenotypic changes

**DOI:** 10.1098/rspb.2017.1295

**Published:** 2017-09-06

**Authors:** Matthew Schrader, Benjamin J. M. Jarrett, Darren Rebar, Rebecca M. Kilner

**Affiliations:** 1 Department of Biology, The University of the South, Sewanee, TN 37383, USA; 2 Department of Zoology, University of Cambridge, Downing Street, Cambridge CB2 3EJ, UK; 3 Department of Biological Sciences, Emporia State University, 1 Kellogg Circle, Emporia, KS 66801, USA

**Keywords:** parental care, cryptic evolution, experimental evolution, co-adaptation, burying beetle, social evolution

## Abstract

Cryptic evolution occurs when evolutionary change is masked by concurrent environmental change. In most cases, evolutionary changes in the phenotype are masked by changing abiotic factors. However, evolutionary change in one trait might also be masked by evolutionary change in another trait, a phenomenon referred to as evolutionary environmental deterioration. Nevertheless, detecting this second type of cryptic evolution is challenging and there are few compelling examples. Here, we describe a likely case of evolutionary environmental deterioration occurring in experimental burying beetle (*Nicrophorus vespilloides*) populations that are adapting to a novel social environment that lacks post-hatching parental care. We found that populations rapidly adapted to the removal of post-hatching parental care. This adaptation involved clear increases in breeding success and larval density (number of dispersing larvae produced per gram of breeding carcass), which in turn masked a concurrent increase in the mean larval mass across generations. This cryptic increase in larval mass was accomplished through a change in the reaction norm that relates mean larval mass to larval density. Our results suggest that cryptic evolution might be commonplace in animal families, because evolving trophic and social interactions can potentially mask evolutionary change in other traits, like body size.

## 1. Introduction

Cryptic evolution occurs when evolutionary change in a trait is masked by a concurrent change in an environmental factor that also influences that trait [1–3]. Cryptic evolution has attracted attention because it provides an explanation for phenotypic stasis in the face of strong and persistent directional selection [3]. Cryptic evolution may also explain phenotypic similarity among geographically distinct populations that differ in their exposure to an environmental variable that influences the phenotype, a phenomenon known as counter-gradient variation [4]. In the best-studied examples of cryptic evolution, a change in an environmental variable such as temperature or population density results in environmental deterioration that obscures evolutionary change in a phenotype [2,3,5,6]. For example, Merilä *et al*. [2] examined whether cryptic evolution might explain why body condition in a population of collared flycatchers (*Ficedula albicollis*) has declined over time despite strong and persistent positive directional selection on this trait. They found that a genetic increase in body condition occurred in this population, but it was probably masked by a decline in the abundance of caterpillars (the main food of nestlings) that was driven by an increase in spring temperatures.

In most cases of cryptic evolution, environmental deterioration is typically attributed to an abiotic factor that changes directionally over time. In some cases, however, evolutionary change in one trait can result in environmental deterioration that masks evolutionary change in another trait [1,3,7]. This has been referred to as ‘evolutionary environmental deterioration’ [3]. For example, an evolutionary increase in a trait such as brood size may lead to environmental deterioration by increasing competition between developing young. This change in the social environment could in turn mask evolutionary changes in an interacting trait such as offspring size that may be under directional selection.

Evolutionary changes in the social environment may be an especially important driver of cryptic evolution in animals with parental care. In these species, offspring often develop in a nursery where diverse social interactions are played out among the family members [8]. Within these nurseries, offspring can compete or cooperate for access to resources and parents can modulate the effect of the offspring's social interactions through direct, or indirect, interventions [8–15]. These social interactions influence the phenotype that offspring attain during development, and can act as a source of selection on these phenotypes [8,16]. In addition, the social environment that arises during parental care is a function of the family members and their underlying genes, meaning that the social environment itself can evolve [17].

Although the social interactions that arise during parental care can generate cryptic evolution, few studies have tested whether evolutionary change in one component of the family environment masks an evolutionary change in an interacting trait. Here, we describe results from populations of burying beetles (*Nicrophorus vespilloides*) that were exposed to experimental evolution. These experimental populations were maintained and evolved for several generations either with or without post-hatching parental care (Control and No Care populations, respectively), but were not otherwise exposed to any form of artificial selection. We tested whether adaptation to a novel family environment involves cryptic change in larval mass, a trait that determines adult size and that is linked to parental performance [18]. We found that the No Care populations rapidly adapted to the removal of post-hatching parental care and that this adaptation involved obvious phenotypic evolution as well as more cryptic phenotypic change. The most obvious signs of adaptation to the removal of parental care were significant increases in breeding success and in larval density (the number of larvae produced per gram of breeding carcass) across the first 13 generations. However, further analyses also revealed cryptic increases in larval mass in the No Care populations. This cryptic increase in larval mass was accomplished through a change in the reaction norm that relates mean larval mass to larval density. Our results provide a likely example of cryptic evolution driven by ‘evolutionary environmental deterioration’ [3].

## 2. Methods

### (a) Study species

Breeding in *N. vespilloides* requires a dead vertebrate, which the parents prepare for their young to feed upon [19,20]. Carcass preparation involves removing the fur or feathers from the carcass, rolling the carcass into a ball, smearing the carcass with antimicrobial exudates, and burying it in a shallow grave. After the larvae hatch, they crawl to the carcass which provides all of the resources the larvae will use to complete their development. Larvae are able to self-feed, but also beg to be directly provisioned by their parents who regurgitate predigested carrion into their mouths. Direct provisioning constitutes the majority of post-hatching care, and is most important within the first 24 h after hatching [21]. Although post-hatching parental care increases larval fitness, *N. vespilloides* larvae can survive without it [21–23].

### (b) Experimental populations

We took advantage of the facultative nature of post-hatching care in *N. vespilloides* and established experimental populations that differed in the amount of post-hatching care larvae receive. In one set of populations (Control), both parents remained with the brood until larval dispersal, 8 days after pairing. This allowed all possible interactions between parents and larvae to be expressed. In the other set of populations (No Care), we removed both parents after they finished preparing the carcass but before their larvae had hatched. This effectively removed all post-hatching parental care.

Wild beetles from four localities were interbred to produce a large, genetically diverse stock population (for details, see the electronic supplementary material). From this stock population, we established two replicate Control populations and two replicate No Care populations. In the Control populations, we allowed both parents to remain with their larvae for the entire larval period. In these populations, we bred an average of approximately 34 pairs of unrelated beetles each generation (electronic supplementary material, table S1). Each pair was placed in a plastic box (17 × 12 × 6 cm) half-filled with damp soil and containing a thawed mouse carcass weighing between 8 and 16 g (see the electronic supplementary material, table S2, for the average carcass mass used in each generation). At larval dispersal (8 days after pairing), we counted the number of dispersing larvae and weighed the entire brood. The dispersing larvae were placed in individual 2 × 2×2 cm cells within a plastic eclosion box (10 × 10 × 2 cm), covered with damp peat, and left to pupate. Newly eclosed adults were given a unique identifying number, housed individually in plastic boxes (12 × 8 × 2 cm), and fed ground beef twice a week until they were bred, 17 days after eclosion.

In the No Care populations, we bred an average of approximately 68 pairs of unrelated beetles each generation (electronic supplementary material, table S1). Note that we bred more No Care pairs each generation to compensate for the higher rate of breeding failures in the No Care populations, and that the average number of successful pairs in each generation was similar between each Control population and its corresponding No Care population (see the electronic supplementary material, table S1). The No Care populations were maintained in exactly the same way as the Control populations, except that we removed both parents 53 h after pairing. This is enough time for the parents to prepare the carcass and for females to complete laying a clutch, but is before larvae hatch [22]. At each generation, breeding pairs were randomly formed within each population, based upon their unique identifying number, with the only condition that males and females assigned to a breeding pair were not siblings (i.e. they were not from the same brood) or first cousins (i.e. their parents were not from the same brood).

We followed the same protocol in every generation with two exceptions. First, in generation four there was a shortage of 8–16 g mice and we had to use mice that were, on average, nearly twice as heavy as those used in the other generations (see the electronic supplementary material, table S2). Second, in generation six we bred all of the No Care pairs with full parental care. This was done to reduce the vertical transmission of mites that had appeared in the previous generation. The fourth generation of all populations and the sixth generation of each No Care population were excluded from all of our analyses.

Over the first 13 generations, we bred 2771 pairs of beetles. For each breeding pair, we recorded breeding success (whether each pair produced at least one dispersing larva), larval density (the number of dispersing larvae divided by the initial mass of the breeding carcass) and mean larval mass (total brood mass at dispersal divided by brood size at dispersal). We focused on each of these measures of performance because they have been shown to be higher with parental care than without [21,22]. In addition, larval density provides information regarding the level of competition between burying beetle larvae [11,24].

### (c) Statistical analyses

We tested whether adaptation to the removal of parental care involved directional changes in breeding success, larval density and the mean larval mass. Each performance measure was calculated at the population level for each generation and the analyses were conducted on these population-level measures of performance. Analysis of larval density only included successful broods (i.e. failed broods were not included as 0s when calculating the mean larval density). We tested for changes in each response variable using a linear model with generation, environment (Control versus No Care) and replicate population as explanatory variables. We initially included the three-way interaction between environment, generation and replicate in each of these models and all two-way interactions involving the replicate. These interactions were never significant, so they were removed from the models.

We performed a second set of analyses to test whether the mean larval mass changed across generations after accounting for the relationship between larval mass and larval density. Because this relationship differs between the No Care and Control populations (see the electronic supplementary material), we analysed each group separately. For the No Care populations, a cubic polynomial best described the relationship between mean larval mass and larval density (see electronic supplementary material). We next tested whether the residuals from this regression changed in a consistent way across generations. To do this, we extracted the residuals from this model and used a linear model to test for the effects of generation and replicate population on residual larval mass, using the mean residual mass for each generation as the independent variable. We included the generation by replicate interaction in the initial model. This interaction was not significant and was removed from the final model. A consistent change in residual mass across generations would indicate a violation of the assumption that residual mass is independent of generation [25]. One biological interpretation of such a violation is that, for a given larval density, larval mass is increasing (or decreasing) across generations.

We performed the same analysis for the Control populations. In the Control populations, a cubic polynomial best described the relationship between mean larval mass and mean larval density (see the electronic supplementary material). We then extracted the residuals from this regression and used a linear model to test for the effects of generation and replicate population on residual larval mass. We included the generation by replicate interaction in the initial model. This interaction was not significant and was removed from the final model.

In the analyses described above, we found that residual larval mass increased across generations in the No Care populations but was independent of generation in the Control populations (see results below). While these results suggest that larval mass increased in the No Care populations, analysis of residuals from linear models can produce biased parameter estimates [25]. We thus examined whether a simple change in the height of the reaction norm relating larval mass to larval density could explain the increase in residual mass that we observed in the No Care populations. To test for such a change, we compared the relationship between mean larval mass and larval density in generation 2 (the first generation in which the parents had developed without parental care) and generation 13 of each No Care population. To limit the number of interaction terms in these models, we analysed each replicate separately. For each replicate, we examined the effect of larval density, larval density^2^, larval density^3^ and generation on the mean larval mass. We initially included interactions between each density term and generation to test whether the shape of the relationship between mean larval mass and larval density differed between generations. These interactions were not significant, indicating that the shape of the relationship between mean larval mass and larval density was similar in generation 2 and generation 13. Consequently, they were removed from the final model. All analyses were performed in R v. 3.3.1 [26].

## 3. Results

**Figure 1.**
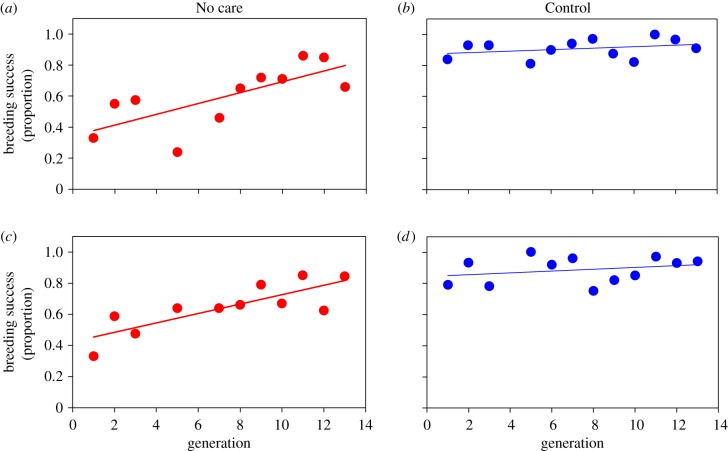
Breeding success (the proportion of pairs producing at least one dispersing larva) in the No Care (red) and Control (blue) populations across 13 generations. The different panels show results for the different replicate populations. Breeding success increased significantly across generations in the No Care populations (*a* and *c*) and remained unchanged in the Control populations (*b* and *d*). Lines are from linear regressions of breeding success on generation for each population.

**Figure 2.**
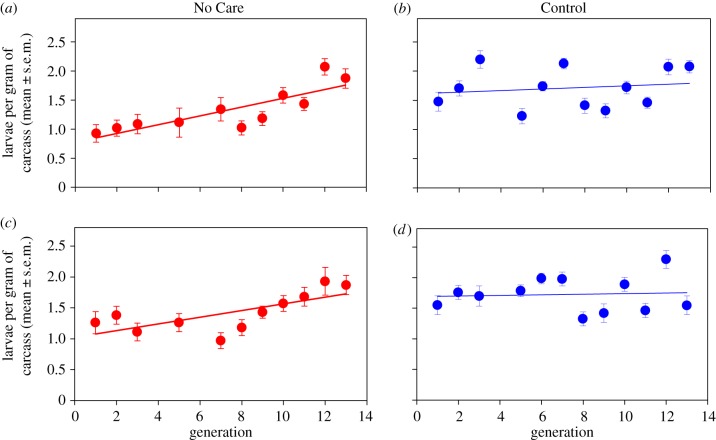
Larval density (mean ± s.e.m.) in the No Care (red) and Control (blue) populations across 13 generations. The different panels show results for the different replicate populations. Mean larval density increased significantly across generations in the No Care populations (*a* and *c*) and remained unchanged in the Control populations (*b* and *d*). Lines are from linear regressions of mean larval density on generation for each population.

Over 13 generations, we observed both obvious and cryptic adaptation to the No Care environment. First, we found that breeding success in the No Care populations increased significantly, nearing the level of breeding success in the Control populations, which remained unchanged (figure 1 and table 1). This increase in breeding success was accompanied by a change in the social environment experienced by larvae in the No Care populations. Specifically, we found that larval density increased significantly across 13 generations in both No Care populations but did not change in a consistent manner across generations in the Control populations (figure 2 and table 1). A similar analysis of the mean brood size (with carcass mass as a covariate) yielded the same qualitative results (see the electronic supplementary material). Previous studies have shown that larval competition in *N. vespilloides* increases with brood size/larval density [11,24]. Thus, our results indicate that as the No Care populations adapted to the removal of care, the social environment experienced by larvae also changed, with larval interactions becoming more competitive with the rise in larval density. As a consequence of increased larval density, we expected to see a decline in average larval mass due to increased competition between larvae for food on the carcass [11,24]. However, we found no significant change in the average larval mass across generations in any of our experimental populations (electronic supplementary material, figure S1; table 1).

**Table 1.** Results of generalized linear models examining the effects of Environment (Control or No Care), Generation, the Environment × Generation and replicate population on Breeding success, mean larval density and mean larval mass.

	breeding success		mean larval density		mean larval mass	
factor	*F*_1,41_	*p*	*F*_1,41_	*p*	*F*_1,41_	*p*
Environment (E)	93.52	<0.00001	21.39	<0.00001	100.41	<0.00001
Generation (G)	24.71	<0.00001	16.07	0.00025	0.16	0.69
E × G	13.62	0.00065	4.14	0.048	1.64	0.21
Replicate	0.15	0.70	1.053	0.31	2.52	0.12

**Figure 3.**
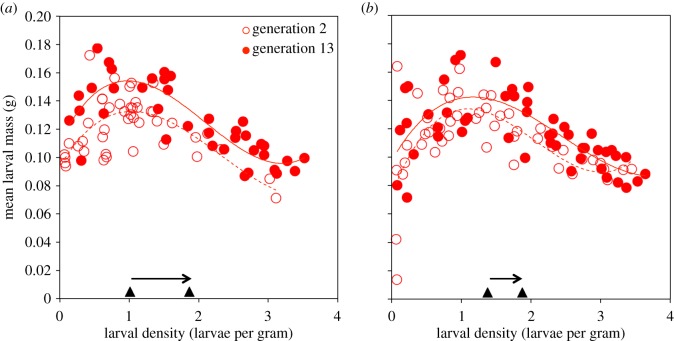
The relationship between mean larval mass and larval density in generations 2 (open circles, dashed line) and 13 (filled circles, solid line) of the No Care populations. Data from replicates 1 and 2 are shown in *a* and *b*, respectively. In each panel, the black triangles and arrow along the *x*-axis illustrates the change in the mean larval density between generations 2 and 13. (Online version in colour.)

Further analyses uncovered evidence of cryptic evolutionary change in larval mass that was concealed by the change in larval density. In the No Care populations, the relationship between mean larval mass (*y*) and larval density (*x*) was described by a cubic polynomial, similar to that seen in previous studies of *N. vespilloides* [11,27] (*y* = 0.10 + 0.069*x* − 0.043*x*^2^ + 0.0061*x*^3^; *F*_3,906_ = 175.2, *p* < 0.00001, *r^2^* = 0.37; electronic supplementary material, figure S2*a*). To uncover cryptic evolutionary change in larval mass, we controlled statistically for larval density by analysing the residuals of the regression of larval mass on larval density. This revealed that the average residual mass increased across generations in each of the No Care populations (generation: *F*_1,19_ = 8.16, *p* = 0.010; replicate: *F*_1,19_ = 0.016, *p* = 0.90; electronic supplementary material, figure S2*b*). Further analyses indicated that the change in residual larval mass across generations was due to a shift in the height of the relationship between the mean larval mass and larval density. For each replicate population, the mean larval mass was greater in generation 13 than generation 2 across the same range of larval densities (figure 3 and table 2). These results are consistent with an increase in the height of the curve relating mean larval mass to larval density in the No Care populations (figure 3).

**Table 2.** Results of analyses comparing the relationship between mean larval mass and larval density in generations 2 and 13 of the No Care populations. Interactions between density terms and generation were not significant and were removed from the models. The two replicate populations were analysed separately.

	No Care A			No Care B		
Factor	Estimate (s.e.)	*F*_1,77_	*p*	Estimate (s.e.)	*F*_1,91_	*p*
Density	0.093 (0.015)	35.28	<0.0001	0.10 (0.018)	13.90	0.004
Density^2^	−0.061 (0.011)	43.32	<0.0001	−0.059 (0.012)	44.48	<0.0001
Density^3^	0.0096 (0.0021)	14.38	<0.0001	0.0.0086 (0.0022)	14.78	0.0002
Generation	0.019 (0.0036)	28.31	<0.0001	0.011 (0.0041)	7.69	0.007
*R*^2^	0.61			0.47		

We found no equivalent evidence for cryptic evolutionary change in the Control populations. Here, too, the relationship between mean larval mass (*y*) and larval density (*x*) was described by a cubic polynomial (*y* = 0.20 – 0.014*x* – 0.018*x*^2^ + 0.004*x*^3^; *F*_1,738_ = 630, *p* < 0.00001, *r*^2^ = 0.63; electronic supplementary material, figure S2*c*). However, unlike in the No Care populations, we found no consistent change in residual larval mass across generations in either of the replicate Control populations (generation: *F*_1,23_ = 0.032, *p* = 0.86; replicate: *F*_1,23_ = 0.014, *p* = 0.91; electronic supplementary material, figure S2*d*). The absence of any directional change in residual mass in the Control populations indicates that the pattern we observed in the No Care populations is not simply a consequence of adaptation to laboratory conditions.

## 4. Discussion

In animals with parental care, the social environment in which offspring develop can be a source of phenotypic variation and an agent of selection [16]. Moreover, because these environments contain genes, they can evolve in response to natural selection [17]. Our results demonstrate that rapid adaptation to an experimental change in one aspect of the social environment experienced during development (namely the absence of post-hatching care) leads to change in another aspect of the environment experienced by larvae (namely larval density, electronic supplementary material, figures 2 and S3). Furthermore, as larval density increased, its influence on offspring phenotype also changed—masking a concurrent increase in the mean larval mass (figure 3). These results indicate that the social environment and the phenotypes of the individuals constituting that environment can each evolve rapidly, consistent with models of interacting phenotypes [17,28]. In addition, our results suggest that concurrent changes in the social environment and the effect of this environment on offspring phenotype can give the appearance of stasis despite significant phenotypic change. This is probably an example of cryptic evolution driven by ‘evolutionary environmental deterioration’ where evolutionary changes in one trait mask concurrent changes in another [3].

Theory predicts that evolutionary environmental deterioration can lead to cryptic evolution. However, detecting this type of evolution is empirically challenging [1,3]. Most demonstrations of cryptic evolution have involved field studies of natural populations where environmental deterioration is caused by changes in climate or population density [2,3,5]. In our study, we can more confidently rule out these potential confounding factors. For example, our populations are maintained in the laboratory where temporal variation in environmental conditions (e.g. temperature, adult food level, carcass mass) is minimized. In addition, because all adult beetles are housed and fed individually, except when they breed, changes in population density are not likely to contribute to phenotypic changes in our populations. Finally, because each No Care population is maintained in tandem with a Control population that breeds on the same schedule, any change in environmental conditions should affect the No Care and Control populations similarly. In our study, however, increases in larval performance were restricted to the No Care populations, indicating that environmental changes that were experienced by both the No Care and Control populations cannot explain our results.

The cryptic increase in larval mass that we observed in the No Care populations appears to be due to a change in the reaction norm that relates larval mass to larval density. Specifically, the reaction norm had a greater elevation in generation 13 than it had in generation 2 (figure 3). This suggests that larvae in the No Care populations have evolved to become more effective at converting the carcass resource into larval mass. How, exactly, has this occurred? We have three suggestions, which are not mutually exclusive. The first possibility is that individual larvae became better at acquiring resources from the carcass without parental help, and more efficient at diverting those resources into growth. This might have involved behavioural, physiological or morphological changes in larvae to increase their ability to extract energy without their parents. Adaptations like this have been observed in *Drosophila melanogaster* populations maintained under crowded, nutritionally stressed conditions [29]. Second, it is possible that there has been a change in some component of pre-hatching parental care that helps larvae to access the resources in the carcass without their parents. For example, No Care parents may create feeding depressions in the carcass earlier than is normal. This might increase the likelihood that larvae can feed on the carcass even when the parents are not there. A final possibility is that increases in larval density enhance the collective ability of larvae to access resources on the carcass, which leads to increased average larval mass. We have previously shown that the ability of *N. vespilloides* larvae to colonize the breeding carcass increases with larval density, presumably because larvae in larger broods work together to chew their way into the carcass [11]. Thus, whereas Hadfield *et al*. [3] suggest that evolutionary stasis in body size could result from ever-increasing competitive ability among siblings for limited resources, our results suggest the same effect could result from increasingly cooperative interactions among siblings in larger broods for resources that have not yet become limiting. Further work is needed to distinguish between the three possibilities outlined above. However, the fact that larval mass was greater across all densities in generation 13 compared with generation 2 suggests that the first or second explanations (involving adaptations in individual larvae or parents) are most likely.

While our results are consistent with cryptic evolution in response to evolutionary environmental deterioration, there are two remaining challenges that we hope to address in future work. First, an unambiguous demonstration of cryptic evolution driven by evolutionary environmental deterioration requires evidence that a genetically based change in one trait has masked a genetically based change in another [3]. For our study, that means genetically based changes in breeding success and larval density would need to mask a genetically based change in the mean larval mass. The experiment we describe here does not allow us to test whether the increases in breeding success, larval density and larval mass have a genetic basis. However, results from a previous study involving separate populations evolving under the same conditions suggest that adaptation to the No Care environment involves genetic changes in breeding success and possibly brood size at dispersal, which is the major determinant of larval density [22].

A second remaining challenge is to identify the specific traits underlying larval performance in the No Care environment and how they have evolved. On the one hand, it is possible that a change in a single larval trait, such as self-feeding behaviour, underlies increases in all measures of breeding performance. On the other hand, separate traits may influence breeding success and larval mass. For example, self-feeding behaviour may influence larval survival, while some other behavioural trait may mediate between-larva interactions, thereby influencing larval mass. It will be especially interesting to examine whether increases in larval mass in the No Care populations have been caused by changes in the behavioural interactions among sibling larvae. Such a change would suggest that indirect genetic effects may play a role in generating cryptic evolution.

Cooke *et al.*'s [1] original model of cryptic evolution focused on clutch size, though they argued that their model could also be applied to traits such as male attractiveness that mediate social interactions between individuals. Animal families are another arena in which social interactions can be a source of selection [8,9,28], and our results indicate that these interactions can generate cryptic evolution. Indeed, cryptic evolution might be especially common in species with parental care because the social environment that arises during care can evolve, and evolutionary change in this social environment can influence the phenotypes that are attained by offspring and can also exert selection on these phenotypes [16]. It is thus surprising that cryptic evolution has been largely ignored in the study of parental care. Understanding why body size (and other traits) evolve so slowly remains a puzzle for evolutionary biology [30]. Perhaps future studies of other species should consider whether trait evolution could be masked by ongoing evolution in the family social environment.

## Supplementary Material

Supplementary Methods and Results

## Data accessibility

Data have been deposited in Dryad (http://dx.doi.org/10.5061/dryad.3kh41) [31].
