# Supplementary material for: Adaptation to a novel family environment involves both apparent and cryptic phenotypic changes
Source: Proc Biol Sci. 2017 Sep 6;284(1862):20171295. doi: 10.1098/rspb.2017.1295 (PMC5597835; doi:10.1098/rspb.2017.1295)
Supplement: Supplementary Methods and Results [file rspb20171295supp1.pdf]

Supplementary Methods and Results

Populations

The experimental populations were established from a pool of 671 individuals descended from 32 pairs of wild beetles collected from four localities in Cambridgeshire, UK (Byron's Pool, Gamlingay, Waresley, and Overall Grove). To minimize the potential for inbreeding, the 32 founding pairs were almost all between locality crosses (the only exceptions were two breeding pairs where both individuals were collected from Byron's Pool). The straight-line distance between these populations is between 2.5 km (between Waresley and Gamlingay) and 20.3 km (between Bryon's Pool and Gamlingay). A recent study of three of these populations (Byron's Pool, Gamlingay, and Waresley) indicates that there are significant population differences at neutral genetic markers between W/G and B but not between W and G (1).

The relationship between mean larval mass and larval density

Previous studies (2, 3, 4) and preliminary analyses indicated that the relationship between mean larval mass and larval density differed between the No Care and Control populations. We thus analyzed each of these groups separately.

For the No Care populations, we began by fitting a cubic polynomial regression of mean larval mass on larval density to the combined data set from both replicates and all generations. We compared the fit of this model to both a quadratic model and a linear model fit to the same data set. These comparisons indicated that a cubic polynomial best describes the relationship between mean larval mass (y) and larval density (x) (Figure S2*a*; $y = 0.10 + 0.069x - 0.043x^2 + 0.0061x^3$; $F_{3, 906} = 175.2$, $P < 0.00001$, $r^2 = 0.37$; $AIC_{cubic} = -4683.6$, $AIC_{quadratic} = -4589.1$, $AIC_{linear} = -4404.4$;).

We used the same approach for the control populations. Here, the relationship between mean larval mass (y) and larval density (x) was best described by a cubic polynomial (Figure S2*c*; $y = 0.1981 - 0.0141x - 0.0177x^2 + 0.004x^3$; $r^2 = 0.64$; $AIC_{cubic} = -3794.4$, $AIC_{quadratic} = -3784.4$, $AIC_{linear} = -3777.3$).

Changes in Brood Size

In addition to testing whether adaptation to the No Care environment involved changes in larval density, we also tested whether there were changes in mean brood size. Brood size in *N. vespilloides* is influenced by a combination of clutch size and larval survival. When parents care for larvae, larval survival is influenced by filial cannibalism that serves to match brood size to carcass size (5). Without parental care, filial cannibalism cannot influence larval survival. We tested for changes in mean brood size at dispersal using a linear model with generation, environment (Control versus No Care), and replicate population as explanatory variables and mean carcass mass as a covariate.

The results of this analysis are nearly identical to the analysis of larval density presented in the main text. We found significant effects of the environment ($F_{1, 45} = 25.65$, $P = 0.021$), the generation by environment interaction ($F_{1, 45} = 5.75$, $P < 0.0001$), and carcass mass ($F_{1, 45} = 8.08$, $P = 0.007$) on brood size at dispersal. There was no difference between replicates ($F_{1, 45} = 1.34$, $P = 0.51$). In both No Care populations mean brood size increased across the first 13 generations

(Figure S3). In contrast, mean brood size in the Control populations did not change significantly across the first 13 generations (Figure S3). The similarity between these results and the analysis of larval density is not surprising given the strong positive correlation between brood size and larval density ($r = 0.95$, n = 46, $P < 0.000001$).

Literature Cited

1. Pascoal S, and Kilner RM. 2017 Development and application of 14 microsatellite markers in the burying beetle Nicrophorus vespilloides reveals population genetic differentiation at local spatial scales. *PeerJ*, doi: 10.7717/peerj.3278

2. Schrader M, Jarrett BJM, Kilner RM. 2015 Parental care masks a density-dependent shift from cooperation to competition among burying beetle larvae. *Evol. Int. J. Org. Evol.* **69**, 1077–1084.

3. Schrader M, Jarrett BJM, Kilner RM. 2015 Using experimental evolution to study adaptations for life within the family. *Am. Nat.* **185**, 610–619.

4. Schrader M, Crosby RM, Hesketh AR, Jarrett BJM, Kilner RM. 2016 A limit on the extent to which increased egg size can compensate for a poor postnatal environment revealed experimentally in the burying beetle, *Nicrophorus vespilloides*. *Ecol. Evol.* **6,** 329–336.

5. Bartlett J. 1987 Filial canabilism in burying beetles. *Behav. Ecol. Sociobiol.* **21**, 179-183.

Table S1. The number of pairs bred (number successful) in each population in each generation of the experiment. The bottom row shows average number of pairs that were bred across all the first 13 generations of the experiment.

Generation	Control 1	No Care 1	Control 2	No Care 2
1	25 (21)	60 (20)	24 (19)	64 (21)
2	30 (28)	80 (44)	30 (28)	80 (47)
3	27 (25)	80 (46)	27 (21)	80 (38)
4	30 (28)	80 (49)	30 (28)	80 (70)
5	27 (22)	80 (19)	30 (30)	75 (48)
6	60 (54)	60 (47)	60 (55)	60 (49)
7	31 (29)	76 (33)	28 (27)	78 (50)
8	34 (33)	72 (47)	36 (27)	79 (52)
9	40 (35)	78 (56)	38 (31)	78 (62)
10	45 (37)	70 (50)	54 (46)	75 (50)
11	29 (29)	50 (43)	29 (28)	47 (40)
12	30 (29)	40 (34)	29 (27)	40 (25)
13	34 (31)	57 (38)	35 (33)	58 (49)
Mean (SE)	34(2.64)	67.92 (3.68)	34.61 (2.97)	68.77 (3.80)

Table S2. Mean (±SE) carcass mass used in each population in the first 13 generations of the experiment.

Generation	Control 1	No Care 1	Control 2	No Care 2
1	10.15 (0.23)	10.79 (0.18)	11.30 (0.30)	11.01 (0.23)
2	12.09 (0.28)	12.95 (0.19)	11.86 (0.32)	11.77 (0.22)
3	12.02 (0.27)	11.67 (0.16)	12.67 (0.59)	11.89 (0.21)
4	23.00 (0.28)	23.19 (0.18)	22.94 (0.29)	24.40 (0.27)
5	11.21 (0.27)	12.06 (0.18)	12.62 (0.30)	12.08 (0.18)
6	12.02 (0.15)	11.92 (0.18)	11.61 (0.24)	11.97 (0.27)
7	11.94 (0.37)	11.26 (0.18)	13.03 (0.63)	12.41 (0.22)
8	10.78 (0.30)	11.14 (0.19)	10.95 (0.23)	10.62 (0.17)
9	10.21 (0.24)	10.55 (0.16)	10.69 (0.26)	10.60 (0.17)
10	10.89 (0.25)	11.23 (0.22)	10.53 (0.18)	10.18 (0.15)
11	12.35 (0.26)	12.29 (0.26)	12.87 (0.19)	11.20 (0.24)
12	11.26 (0.22)	11.09 (0.19)	10.44 (0.26)	10.04 (0.23)
13	10.80 (0.26)	10.63 (0.18)	11.80 (0.30)	10.71 (0.24)

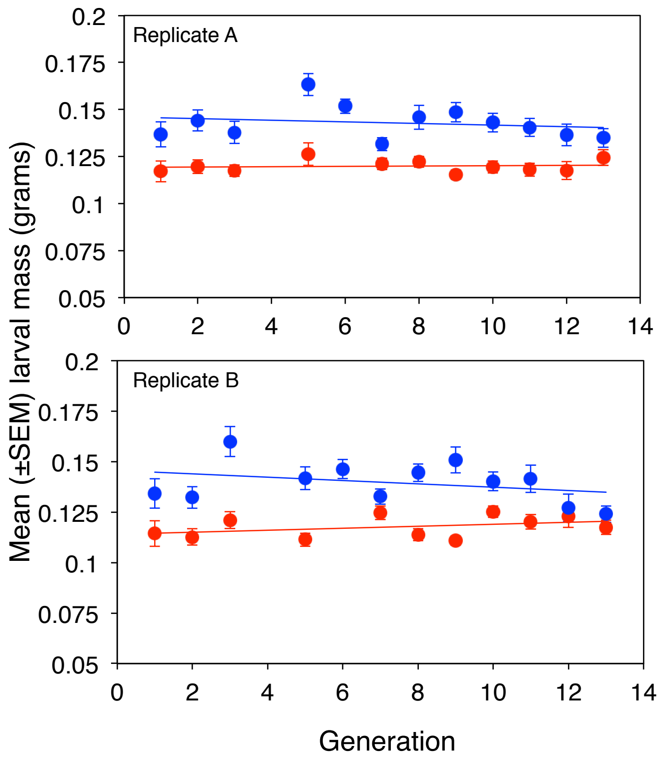

Figure S1. Mean larval mass (± SEM) in the No Care (red) and Control (blue) populations across 13 generations. Data from the different replicates are shown in different panels.

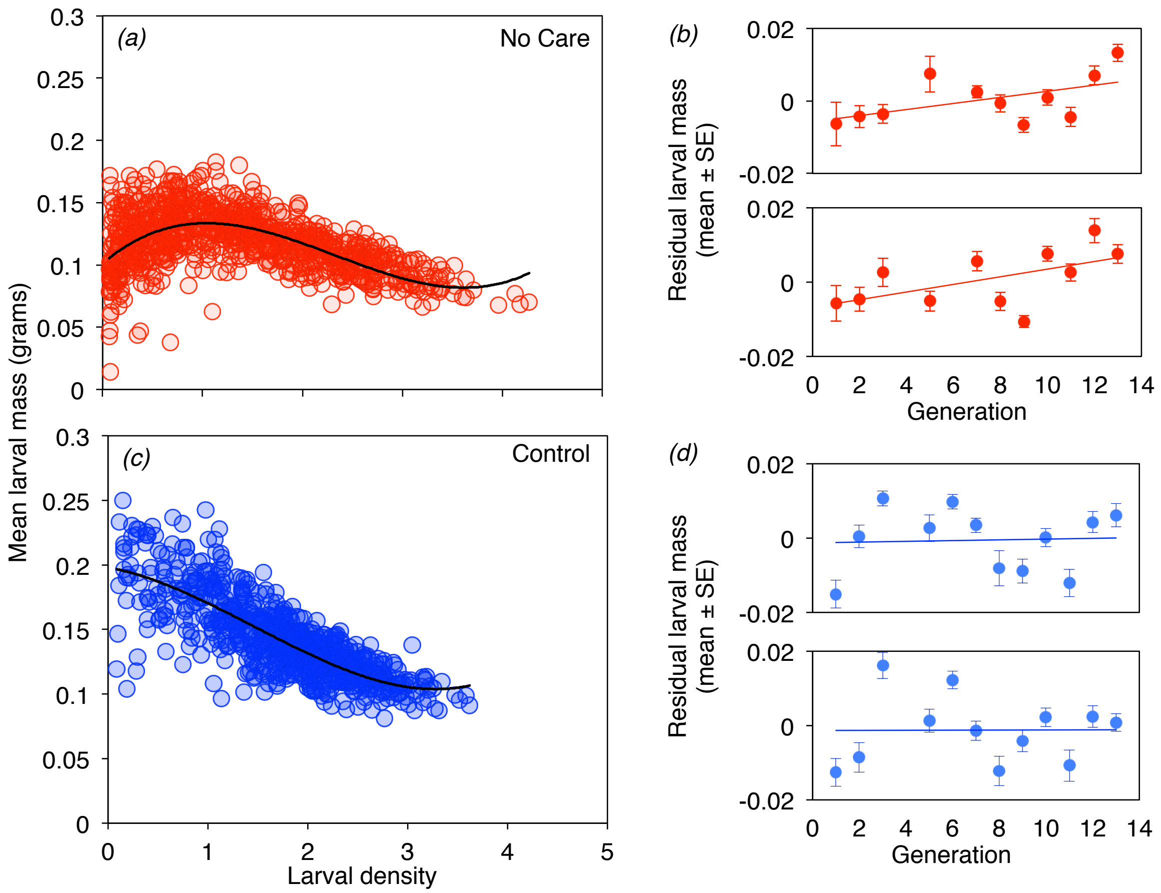

Figure S2. The relationships between mean larval mass and larval density, and the change in residual larval mass across generations in the No Care and Control populations. *(a)* The relationship between mean larval mass and larval density in the No Care populations, pooling across replicate populations and generations. The line is from the regression analysis described in the text. (*b*) Residual larval mass (mean ± SEM) in the No Care populations across 13 generations. Residual larval mass was extracted from the regression in (*a*) and the different panels are from the different replicate populations. (*c*) The relationship between mean larval mass and larval density in the Control populations, pooling across replicate populations and generations. The line is from the regression analysis described in the text. (*d*) Residual larval mass (mean ± SEM) in the Control populations across13 generations. Residual larval mass was extracted from the regression in (*c*) and the different panels are from the different replicate populations.

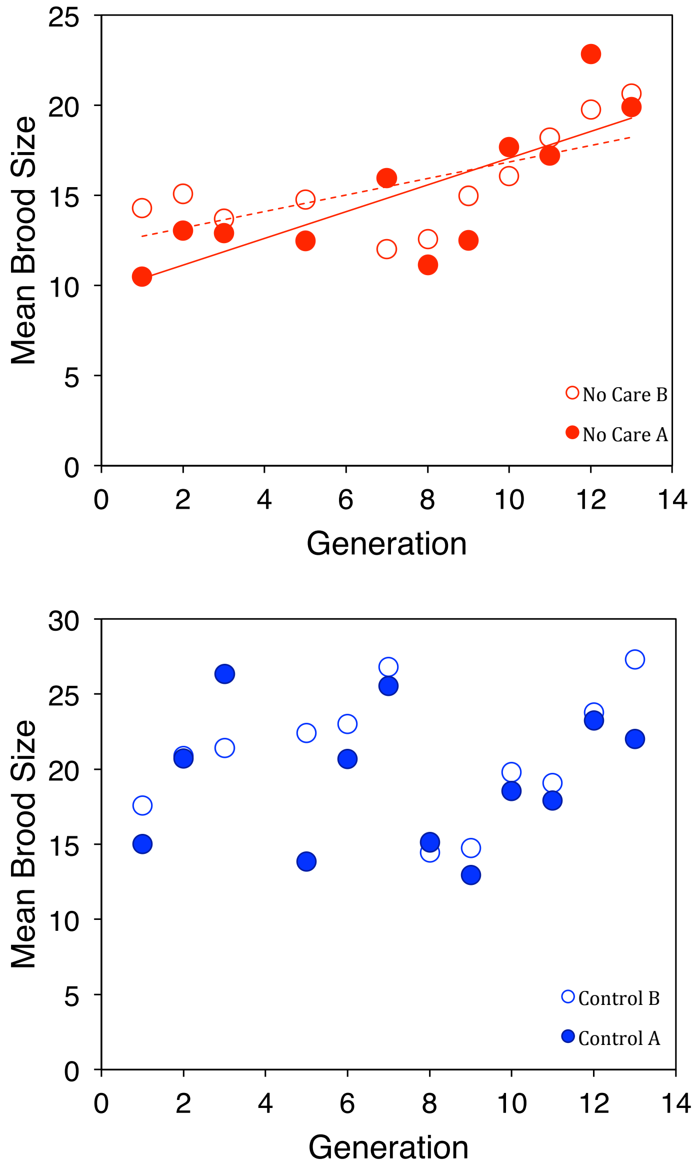

Figure S3. Mean brood size in the No Care (red) and Control (blue) populations across 13 generations. Open and solid symbols represent different replicate populations. Mean brood size increased significantly across generations in the No Care populations and remained unchanged in the Control populations. Lines are from linear regressions of mean brood size on generation for each No Care population.
